# On Suicide—Verdicts of Felo-de-se


**Published:** 1850-01-01

**Authors:** 


### Art. III.—ON SUICIDE.—VERDICTS OF FELO-DE-SE.*

The late and much lamented death of the distinguished and rising surgeon, Mr. Morton, has again directed public and professional cognisance to the subject of suicide. With the view of exposing many of the fallacies existing in the public mind in connexion with this subject, and of directing professional and general attention to the importance of watching with earnest care the incipient indications of that condition of brain and nervous system, which so often leads to the act of self-destruction, the editor of this Journal published a letter on Suicide in an influential and ably conducte

* The Report of the inquest on the death of the late Mr. Morton.

morning paper.* The subject is of such vast importance, that we consider no apology necessary for recapitulating the facts and observations contained in the article referred to.

We have for a long period of our professional life devoted much attention to the consideration of that form of morbid mind accompanied with the suicidal impulse, and we have always been under the impression that the deranged feeling which so often leads to the commission of self-destruction has not been investigated by medical psychologists in a manner commensurate with its vast importance. We consider that great and incalculable advantages are likely to accrue from the dissemination of sound views in relation to this matter. The public mind cannot be too much enlightened as to the real causes of disease, nor can any evils arise from a general acquaintance with the first manifestations of disordered action, be it of body or mind, or with the principles of medical treatment. Let us illustrate this observation by referring to the subject of suicide. It is the prevalent opinion among persons ignorant of the science of psychology and pathology, that the desire of self-destruction is, in the majority of cases, a *mental* act, unconnected with a disturbed condition of the *bodily* functions, and incurable by any process of medical treatment; that the depression of mind which is so generally associated with the suicidal tendency is an affection of the mind *per se*, the physical organization having no direct connexion with what is termed the *spiritual* impulse. This, we regret to say, is the generally received opinion, even among men of great attainments and of a high order of intellect. This metaphysical view of the matter is fraught with much mischief, and we have no doubt has led to the neglect of cases of actual insanity, and to the sacrifice of many valuable lives. It is a matter of the highest moment that the public mind should be undeceived upon this point. Right views on this subject ought to be generally diffused—it is of consequence to establish the belief that the suicidal idea is *almost generally* connected with a morbid condition of the mind, and is often the only symptom of such an affection—that it is, with a few exceptions, universally associated with physical disorder disturbing the healthy balance of the understanding—and that this bodily affection, which is, in nine cases out of ten, the *cause* of the mental irregularity, is easily curable by the judicious application of the principles of medical science. The tendency of the spiritual or metaphysical view of the question is to create a distrust in remedial measures; and the poor man who is struggling against an almost overpowering desire to destroy himself is either induced to run to

**The Morning Chronicle.*

the clergyman or to his prayer-book for assistance, or to neglect entirely his lamentable condition under the belief that he is literally placed beyond the reach of curative agents, and that the only remedy for his mental suffering is death.

If a person in this unhappy state of mind is induced to believe that his mental despondency is but a *consequence* or *effect* of a disturbed condition of certain organic functions, influencing either directly or indirectly the natural and healthy operation of the brain and nervous system, and giving rise to perverted ideas—that his malady is curable—he may be induced to avail himself of the means which science has placed at the disposal of the physician, and thus be protected against his own insane impulses.

Where no disease is suspected, no remedy will be sought. Tell a man who has attempted to slay himself, that he is perfectly sane—that his judgment is sound—that his will is not perverted—that the impulse which drives him to the commission of suicide is not associated with any deviation from corporeal health,—and you inculcate ideas not only fallacious, but most pernicious in their character and tendency. We might with as much truth tell a person playing with a lighted taper at the edge of a barrel of gunpowder, that his life is not in jeopardy, as to say to a person disposed to cut his throat, blow out his brains, poison or hang himself, that he is in the perfect enjoyment of health, and requires no moral or medical treatment. It may be laid down as an indisputable axiom, that in every case of this kind, bodily disease may, upon a careful examination, be detected. We never yet saw a case where a desire to commit suicide was present, in which there was not corporeal indisposition. Instead of inculcating the notion that this morbid propensity is independent of material lesion, it should be our constant endeavour, and in fact it is our duty, to establish the necessity of obtaining medical advice directly the idea of self-destruction takes possession of the mind. We have seen the most happy results ensue from adopting such a course. "To know ourselves diseased is half our cure," is a wise maxim when applied to this subject. It ought to be our object to persuade those so unhappily afflicted, that the unnatural propensity is but the consequence of physical ailments, which are amenable to those medical principles which direct the practitioner in the treatment of other diseases. We ought to diffuse through society generally the notion that it is a matter of essential importance, that the patient whose mind is haunted by the idea of suicide should instantly subject himself to medical treatment. He should be informed that his feelings are but the effect of physical disorder, which can be easily removed if he will confide in the judgment of a medical practitioner. We have seen, in cases of this description, a strong and apparently uncontrollable suicidal impulse yield to a few simple remedies. The physician, when consulted in a case of suicidal mania, should carefully ascertain whether there be congestion of the brain or abdominal viscera. The latter affection is often present in this class of patients. The condition of the liver should also be inquired into. In the majority of cases that organ will be found in a morbid condition. Much may be done by medical treatment in cases of insanity attended by this symptom.

The practitioner should endeavour to obtain the confidence of the patient. It is often possible to reason or to frighten a person out of the desire to commit self-destruction. Suicides are generally cowards. The very act of suicide implies the absence of courage. If the medical man, when consulted in these cases, acts with firmness and resolution, he may often succeed in turning the current of morbid thought. We were once in the presence of a person who had attempted suicide. A friend of the party said to the patient sneeringly, "Pooh, you commit suicide ? you have not the courage to do it—it is all nonsense and bravado; take this knife and cut your throat." Although we questioned the propriety of thus addressing the individual referred to, it had nevertheless a good effect: he never again attempted his own life.

Dr. Darwin states that a gentleman, being apprehensive that his friend meditated self-destruction, obtained from him a promise that he would not do so before four o'clock the next day. He fulfilled his promise, but blew out his brains as soon as the hour was expired. A patient evidently bent on suicide, asked M. Falret for razors to shave himself with. Trusting to his knowledge of the case, M. Falret complied. This physician observes, "The patient, after having shaved, looked at me, and observing that I remained tranquil, he exclaimed, 'How well you know me !' From this time I had all his confidence." It is important to ascertain the presence of the early symptoms of suicidal mania; for in the premonitory stage, medical and moral treatment may be had recourse to with very great advantage. The experienced physician can easily detect the existence of suicidal mania. There is in these cases a peculiar expression of countenance, which constitutes an important diagnostic sign.

For some period before the individual manifests a morbid propensity to sacrifice his own life, the mind may have dwelt upon the idea of self-destruction. In some cases the impulse is apparently sudden in its development; but it will commonly be found, upon inquiry, that the notion of suicide has been haunting the imagination for a considerable time before the act is perpetrated or attempted. When the mind has been directed or engaged in a particular train of morbid contemplation for any length of time, the face assumes a peculiar cast, which is present in no other state of mental feeling. The practitioner who has had any experience in these cases, can easily detect the existence of the suicidal propensity. With the view to the prevention of self-murder, how important it is that the physician should make himself practically acquainted with this indication. Parkman relates that Lord M. having requested Stuart, a distinguished artist in London, to paint a portrait of his brother, a captain in the army,—he did so with great accuracy. When Lord M. saw the portrait, he exclaimed, "This is not the portrait of my brother,—it is the *portrait of a madman*." The painter requested another sitting; his Lordship saw the painting again, when he observed, "*My brother appears more mad than before!*" Three weeks after the Captain blew out his brains!

It is not difficult to conceive the intense agony of the mind in which the idea of self-destruction is constantly uppermost, and when the most powerful of instincts—viz., that of self-preservation—is mastered. The struggle—and a fearful one it often is—between a stern sense of religious and social duty, and a highly-wrought sensitiveness of mind and an overwhelming impulse to suicide, has been known to be of months', of years', duration; the unhappy individual being left a prey to his own morbid yearnings—nothing, literally nothing, being done to remove the physical derangement causing the mental disturbance. It is a rare occurrence for a suicide to take place in which the evidence before the coroner's court does not establish that, for some period *before* the act was committed, "great mental depression" was observed, "the person was incapable of exercising continuity of thought," "was guilty of deviations from his natural course of life," "manifested irritability of temper," "quarrelled with his family or friends," "neglected his business," became "irregular or intemperate in his habits," was "inattentive to his personal appearance," exhibited "great caprice of temper." We appeal to the recollection of our readers whether we are not correct in asserting that evidence of this character is generally adduced at the inquiries instituted by the coroners in cases of suicide? Occasionally an instance occurs which baffles all our psychological knowledge, and in which no apparent cause for the suicidal feeling can, on the most careful scrutiny, be detected. A gentleman, happy in his domestic relations, in the possession of considerable property, loved, admired, and respected by a large circle of private friends, was sitting at the head of his dinner table, having a few select companions as his favoured guests. He had given no previous indication of bodily or mental disease. During dinner he was noticed to be more than usually cheerful and vivacious. He had lost none of his wonted brilliancy of wit or smartness of repartee. Before the repast concluded, the gentleman in question apologized for leaving the table abruptly. He retired suddenly to his own room, went to his razor-case, held his head over a bason, and deliberately cut his throat! The announcement of the dreadful event was made at the moment when his chosen and convivial friends were in the act of drinking his health! We have in our private case-book the particulars of several instances of this kind, inexplicable and subtle in their character, but the one we have cited is sufficient to illustrate our view of the subject. These cases are exceptions to the general rule; and it is essential that this view of the matter should be generally entertained. It ought never to be forgotten that the disposition to suicide may be the only sign present that indicates the existence of disturbed mind; that the commission of suicide is often the *overt* act of insanity; and that when the feeling is present, the individual himself may feel assured that his health is out of order, and that he requires the aid of the physician, as well as of the metaphysician and clergyman. How indisposed are relatives and friends to believe the fact that the mind is in an unsound state, even in cases where the disorder is most obvious and apparent! A gentleman holding a high public situation manifested for some weeks great depression of spirits, but he was not considered sufficiently ill to justify the family in summoning to his aid their ordinary medical adviser. One day he was found suspended by the neck, was fortunately cut down before vitality was extinguished, was placed in his carriage, and taken to the house of a physician. It was soon ascertained that the poor man's bodily health was in a sad condition. By his own confession he had not closed his eyes for *six* days or nights! He recovered, and is at this moment exercising his official duties in the full enjoyment of "*mens sana in corpore sano.*" It is an occasional occurrence for persons labouring under great disturbance of the general health, accompanied with a morbid desire to destroy life, to seek advice from experienced medical men. These cases, if subjected to treatment at the early period of the malady, are generally curable. We say this to comfort those (and they constitute, we fear, a large class)

who, from erroneous views, are led to consider that the door of hope is for ever closed against them.

We have known the suicidal idea completely dispelled by the application of a few leeches to the head. Purgatives judiciously and appropriately administered often act beneficially in these cases. The various preparations of *opium,* if given with judgment, and in cases *where the suicidal impulse is not dependent upon or connected with congestion of the brain,* frequently act like a charm in tranquillizing the nervous system. The cold shower bath, warm bathing, seclusion from excitement, issues, are all excellent remedies in some cases of suicidal mania.

The question of the propriety and justice of verdicts of *felo de se,* has again been mooted in one of our courts of law. In the Bail Court, before Mr. Justice Patterson, Sir F. Thesiger was instructed to move for a *certiorari* to bring into the court an inquisition taken by the coroner for the borough of Leeds, and also the depositions. The inquisition was held on the body of a person of the name of Mary Ann Waley, and a verdict was returned of *felo de se,* the object being to traverse the inquisition; but at the same time he thought it right to state that, when the inquisition was brought into this court, if he found any substantial defects, he should wish to have the opportunity of taking advantage of them. The circumstances of this case were of the most extraordinary and mysterious character. Mary Ann Waley was the daughter of a very respectable person residing in London, and on the 21st of June last, she married Mr. Waley, her husband, who was a wool dealer, residing at Holbeck, in the neighbourhood of Leeds. She was twenty-seven years old, and he was thirty, and they were married with the entire approbation of the parents of the lady. After the marriage, they proceeded first to Leamington, and afterwards to Scarborough, and from both these places letters were written by the wife to her sister, expressing, in the most artless and simple way, her happiness in her new state. They arrived at home on the 2nd of July, and on the 7th of that month a letter was written to her sister, which he would not trouble his lordship with, but it was written precisely in the same strain as all the others. There was nothing exaggerated, but it was a most simple and artless account of her own happiness, and ending in this way:—"It has just struck 5 o'clock, and as my dearest William makes his appearance about that time, I must draw to a close and make ready for his tea; so, dearest Susanna, with our united love, I remain your affectionate and happy sister." On the 9th of July she was seen as late as half-past 11 o'clock in the morning in perfect health and spirits; she was of a very cheerful disposition, of a quiet, well-regulated, and religious mind. She had breakfasted with her husband that morning, and they had parted on the most affectionate terms—the husband going to his daily business, and in five minutes after she had been seen in the state he had described, she was found stretched upon the floor of her bedroom, with her throat cut from ear to ear, and with a razor between the finger and thumb of her left hand. An inquest was held, and upon the direction of the coroner, under these circumstances, the jury were almost compelled to find a verdict of *felo de se.* The coroner's direction, which was sworn to, was of a most extraordinary kind. He said, "Anything leading to show how deceased came by her mortal wound was involved in deep mystery. According to the evidence of Mr. Price, the wound might have been self-inflicted or not, and no just inference could be drawn. However, had it been done by some other person, struggling would have been expected, and it was probable, but not certain, that the cut would have been irregular. That she might have done it, as Mr. Price put it, involved so much doubt, that they could not come, upon his single testimony, to any definite conclusion; supposing that she would do it from the impulse of the moment, it appeared singular that she should use the left hand to the left side of the neck, as the other would have been exerted more readily and more powerfully. But the jury must respect the medical testimony, in stating that it was possible for deceased to have inflicted the wound even in the manner described. There were no grounds for suspicion against any one. The servant girl's evidence was corroborated, and therefore there were no grounds for supposing that she was an agent in the death of her mistress. Was it possible, in the interval spoken of, for any person to have entered the house? This he could not determine, as persons had been so officious and had cleared all away before the jury or any other person capable of forming an opinion on the matter had seen the place. No inference could be drawn from Mr. Kerr's statement, as he was so confused. Looking to the probabilities, it was exceedingly probable that the deceased had done the act herself; but on the other hand it was quite possible that a stranger might have done it, and they knew that murders were sometimes committed without the slightest apparent motive. If they had evidence to show that deceased had exhibited eccentricities, or been easily excited, it would then have favoured the assumption that insanity had supervened, and would have been strong evidence that she had destroyed herself. But she was perfectly sane at half-past 11 o'clock; and if the jury should be of opinion that deceased destroyed herself, the next thing to consider was the state of her mind at the time. It was painful to him to say that he could not see any possibility of her insanity at the time when this occurred; he had thought the matter carefully over, and with the evidence before him he could not reconcile himself to such an opinion. It would be erroneous to say that because persons destroyed themselves they were not of sane mind. In this instance —and it rendered the case more remarkable—they had the strongest evidence, up to five minutes before death, of her sanity. They had better find a verdict of *felo de se*, than, by giving an open verdict, throw a suspicion upon any party, which might, however unjust, endure for the remainder of life." Under this strange direction the jury found the verdict of *felo de se* after three hours' deliberation. It being clear that no suspicion could have attached to any one, if the jury had found the verdict that, if she destroyed herself, it must have been under some sudden attack of madness for which the human mind was not prepared, that would have been a proper verdict, as all knew that such awful visitations had taken place at different times; but, under this direction of the coroner, the jury had returned the verdict of *felo de se*. The family of this lady were extremely anxious to remove this stain upon her memory, and they felt a strong desire that she should have Christian burial; he therefore applied for a *certiorari* to traverse the inquisition; at the same time he would tell his Lordship that if he found the inquisition defective he should take advantage of it.

The judge consented to the application. We are not prepared to assert, as our opinion, that the commission of suicide *is invariably the act of an insane mind;* but we maintain, whenever the tendency to self-destruction exists, and a person lays violent hands upon his own life, that the presumption is strongly in favour of the suicide being the consequence of derangement of mind. We know how difficult it often is to prove the fact of insanity in cases about which we entertain no doubt. Many persons are driven to suicide whilst under the influence of a delusion known only to themselves; which delusion, it has been established, has haunted them for years. The coolness, self-possession, cunning, and cleverness which many manifest, just prior to the commission of self-destruction, is no argument in favour of the presence of *sanity*. The most extraordinary power of control over the thoughts and actions are consistent with the existence of suicidal mania.

Again, we have no right to infer the absence of insanity, because we have it in our power to trace the act of suicide to a real, and, perhaps, its probable cause. A man is exposed to some great mental excitement, reverse of fortune, accession of wealth, or is accused of the commission of some terrible crime; and under the influence of one of these exciting causes, he sacrifices his life. The disposition of juries, under such circumstances, is to bring in a verdict of *felo de se;* but we consider such a course unjustifiable. Who can define what *degree* of mental perturbation is necessary so to disturb the balance of the mind, as to deprive the person of all *sane* control over his thoughts and actions? If our brains were organized according to a given standard, and we were all on a par as to intellectual vigour and nervous energy, then we might safely predicate the *results* following exposure to certain states of the moral atmosphere. But for wise reasons the nervous apparatus is very dissimilarly organized in various individuals, and an amount of mental excitement which would overturn many minds, could be borne with impunity by others. This fact—and it forms an important element in the argument—should never be lost sight of in considering the question before us. The majority of cases of suicide which take place in the middle ranks of life, arise from an over-taxation of the functions of the brain. It is useless to cavil about words, or to conceal a valuable physiological truth in obedience to the prejudice of weak and ignorant minds. As physiologists, we are compelled to consider mental phenomena as a function of nervous matter. We do not pretend to explain the nature of this union—we consider it as a matter of demonstration. Admitting this truth, it is not difficult to understand why an excessive straining of the functions of the brain should frequently derange the operations of the mind. Great anxiety—an undue application to business, requiring much mental exertion—the excessive excitement of mind, often necessarily concomitant with speculations connected with great fluctuations in the value of property—all tend,—most remarkably tend,—to lay the foundation for insanity, and to induce the suicidal disposition. The brain, in these cases, is overworked; its peculiar powers or functions are called into unhealthy exercise, and derangement of ideas, or in other words insanity, is, in certain organizations, the inevitable consequence.

It cannot be too generally known that disturbed *thought* (quite irrespectively of suicidal feeling) is as much a symptom of mischief (we do not say *organic*) of some kind going on in the *brain* as severe pain of one of the joints of the foot, accompanied with a swollen condition of the part, is evidence of an attack of the gout. All sudden and prolonged deviations from the natural disposition, thoughts, character, temper, feelings, affections, and habits, should awaken grave suspicion in the person's own mind, and lead him to ask himself the question, "Is there not something wrong with my bodily health, interfering with the smooth and normal current of my thoughts and feelings?" It is better to be too much upon our guard against the *first inroads* of deranged mind than to wilfully overlook and neglect, as is, alas! often the case, the glaring evidences of disordered intellect.

But to return to the consideration of verdicts of *felo de se.*

The act of suicide ought not to be considered as a crime in the legal definition of the term. It is not an offence that can be deemed cognizable by the civil magistrate. To punish suicide as a crime is to commit a solecism in legislation. The unfortunate individual, by the very act of suicide, places himself beyond the vengeance of the law; he has anticipated its operation; he has rendered himself amenable to the highest tribunal—viz., that of his Creator; no penal enactments, however stringent, can affect him. What is the operation of the law under these circumstances? A verdict of *felo de se* is returned, and the innocent relations of the suicide are disgraced and branded with infamy, and that too on evidence of an *ex-parte* nature. It is unjust, inhuman, unnatural, and unchristian, that the law should punish the innocent family of the man who, in a moment of frenzy, terminates his own miserable existence. It was clearly established, that before the alteration in the law respecting suicide, the fear of being buried in a cross-road, and having a stake driven through the body, had no beneficial effect in decreasing the number of suicides; and the verdict of *felo de se,* now occasionally returned, is productive of no advantage whatever, and only injures the surviving relatives.

When a man contemplates an outrage of the law, the fear of the punishment awarded for the offence may deter him from its commission; but the unhappy person whose desperate circumstances impel him to sacrifice his own life can be influenced by no such fear. His whole mind is absorbed in the consideration of his own miseries, and he even cuts asunder those ties that ought to bind him closely and tenderly to the world he is about to leave. If an affectionate wife and endearing family have no influence in deterring a man from suicide, is it reasonable to suppose that he will be influenced by penal laws?

If the view which has been taken of the cause of suicide be a correct one, no stronger argument can be urged for the impropriety of bringing the strong arm of the law to bear upon those who court a voluntary death. In the majority of cases, it will be found that some heavy calamity has fastened itself upon the mind, and the spirits have been extremely depressed. The individual loses all pleasure in society; hope vanishes, and despair renders life intolerable, and death an apparent relief. The evidence which is generally submitted to a coroner's jury is of necessity imperfect; and although the suicide may, to all appearance, be in possession of his right reason, and have exhibited at the moment of killing himself the greatest calmness, coolness, and self-possession, this would not justify the coroner or jury in concluding that derangement of mind was not present.

If the mind be overpowered by "grief, sickness, infirmity, or other accident," as Sir Matthew Hale expresses it, the law presumes the existence of lunacy. Any passion that powerfully exercises the mind, and prevents the reasoning faculty from performing its duty, causes temporary derangement. It is not necessary in order to establish the presence of insanity to prove the person to be labouring under a delusion of intellect—a false creation of the mind. A man may allow his imagination to dwell upon an idea until it acquires an unhealthy ascendancy over the intellect, and in this way a person may commit suicide from an habitual belief in the justifiableness of the act. If a man, by a distorted process of reasoning, argues himself into a conviction of the propriety of adopting a particular course of conduct, without any reference to the necessary result of that train of thought, it is certainly no evidence of his being in possession of a sound mind. A person may reason himself into a belief that murder, under certain circumstances not authorized by the law, is perfectly just and proper. The circumstance of his allowing his mind to reason on the subject is a *prima facie* case against his sanity; at least it demonstrates a great weakness of the moral constitution. A man's *morale* must be in an imperfect state of development who reasons himself into the conviction that self-murder is under any circumstances justifiable.

We dwell at some length on this subject, because we feel assured that juries do not pay sufficient attention to the influence of passion in overclouding the understanding. If the notion that in every case of suicide the intellectual or moral faculties are perverted, be generally received, it will at once do away with the verdict of *felo de se.* Should the jury entertain a doubt as to the presence of derangement, (and such cases may present themselves,) it is their duty, in accordance with the well-known principle of British jurisprudence, to give the person the benefit of that doubt; and thus a verdict of lunacy may be conscientiously returned in every case of this description.

